# Pathogen genome enrichment sequencing to support a city-wide Legionnaires’ disease outbreak investigation

**DOI:** 10.1099/mgen.0.001836

**Published:** 2026-09-28

**Authors:** Karolina Mercoulia, Jake Lacey, George Taiaroa, Sally Dougall, Francesca Azzato, Stuart Adcock, Maryza Graham, Evelyn Wong, Norelle Sherry, Timothy P. Stinear, Benjamin P. Howden, Andrew H. Buultjens, Torsten Seemann

**Affiliations:** 1 Microbiological Diagnostic Unit Public Health Laboratory, The University of Melbourne, at the Peter Doherty Institute for Infection and Immunity, Melbourne, Victoria, Australia; 2 Department of Microbiology and Immunology, The University of Melbourne at the Peter Doherty Institute for Infection and Immunity, Melbourne, VIC, Australia; 3 The Centre for Pathogen Genomics, The University of Melbourne, Melbourne, Victoria, Australia; 4 Department of Infectious Diseases, The University of Melbourne at The Peter Doherty Institute for Infection and Immunity, Melbourne, Victoria, Australia; 5 Victorian Infectious Diseases Reference Laboratory, The Royal Melbourne Hospital at The Peter Doherty Institute for Infection and Immunity, Melbourne, Victoria, Australia; 6 Health Regulator Branch, Department of Health, Melbourne, Victoria, Australia; 7 Community and Public Health Division, Department of Health, Melbourne, Victoria, Australia

**Keywords:** enrichment sequencing, hybridization capture, *Legionella*, outbreak investigation, public health, whole-genome sequencing

## Abstract

Pathogen genomics can provide high-confidence source attribution for epidemiological investigations of Legionnaires' disease (LD) outbreaks. However, the utility of culture-based genomics can be undermined by long lead times for *Legionella pneumophila* culture or a lack of isolates when dealing with non-culturable bacteria or low-biomass specimens. Here, during a large 2024 LD outbreak in Melbourne, Australia, we compared direct specimen, hybridization-based enrichment sequencing with traditional culture-based whole-genome sequencing of 18 clinical and 5 environmental *L. pneumophila* specimens. Informed by the *L. pneumophila* global population structure, the method used 28,061 RNA bait hybridization probes to enrich >170,000 known genome-wide informative SNP sites directly from clinical and environmental specimens. Genome enrichment sequencing detected *L. pneumophila* with high sensitivity, achieving genome-wide coverage at 100 c.f.u. ml^−1^. Using sequence enrichment data with phylogenomic and machine learning approaches, we identified that a single cooling tower was likely responsible for the outbreak. Genome enrichment sequencing has the potential to enable faster and more comprehensive source tracking, thereby enhancing public health responses to outbreaks of environmental pathogens, permitting earlier, more confident interventions.

**Impact Statement**

Legionnaires’ disease (LD) is a bacterial infection caused by *Legionella* species with *Legionella pneumophila* responsible for ~85–90% of cases worldwide annually. Infection occurs by inhalation of contaminated aerosols, with outbreaks often associated with cooling towers. As aerosols can spread over long distances, outbreaks can have high case numbers and impact large areas of urban populations. Rapid identification of the pathogen source is key to stopping outbreaks. Here, we developed an advanced method for rapidly extracting pathogen genomic information directly from clinical and environmental specimens. The research is significant because the technique was used to rapidly confirm the environmental source of a large LD outbreak that occurred in Melbourne, Australia in 2024. These methods can potentially be implemented in other laboratories performing real-time *Legionella* outbreak investigations on environmental and clinical sources to decrease time to outbreak source detection and directed public health interventions.

## Data Summary

Sequence metadata and accession numbers from all *Legionella* genomes included in analyses in this study are available in Table S1 and whole-genome sequencing data can be accessed at the NCBI Sequence Read Archive (BioProject PRJNA941385).

Details of the enrichment sequencing reads, and their source are available in Table S2 and can be accessed at the NCBI Sequence Read Archive (BioProject PRJNA1426987). Read files generated from clinical human specimens were filtered to remove human host reads prior to deposition to comply with ethics approval.

## Introduction

*Legionella* spp. are Gram-negative bacteria commonly found in aquatic environments. There are two different clinical syndromes attributed to the genus [1]. The most important of these is Legionnaires' disease (LD), often caused by *Legionella pneumophila*, specifically serogroup 1 (LpSG1). LD is an acute severe pneumonia, with an incubation period of 2–14 days. The most common symptoms are fever, a non-productive cough, headaches, myalgia, rigours, dyspnoea and diarrhoea [2]. The second clinical syndrome is Pontiac fever, an acute febrile illness with no accompanying pneumonia that does not generally require treatment.

The genus *Legionella* comprises 69 validly described species as published in the List of Prokaryotic names with Standing in Nomenclature database (https://doi.org/10.83108/rn.515933), of which ~30 have been associated with human infections [3, 4]. *Legionella* are widely distributed in the environment and routinely isolated from aquatic environments such as lakes, rivers, creeks and other bodies of water, and are also present in soils [1]. *Legionella* are also often found in man-made water systems such as air conditioning cooling towers – a common source of infections and outbreaks [5]. *Legionella* persist and replicate within amoebae [1, 2]. The amoebae form biofilms which harbour *Legionella* and can protect the bacteria from some water treatment and disinfection methods. Transmission occurs via inhalation of aerosols produced by contaminated water systems. Human aerosol exposure rates and frequency, along with the virulence and concentration of *Legionella* species involved, are all key determinants of disease [1]. Individual factors that increase susceptibility to *Legionella* disease include co-morbidities such as chronic lung and heart disease, smoking, older age, being male and immunosuppression [1, 5].

LD is often diagnosed using a urinary antigen test in a clinical microbiology setting, with rapid turnaround times offset by lower sensitivity. To improve diagnostic sensitivity, nucleic acid amplification tests (NAATs) from respiratory specimens are often additionally performed for clinical diagnosis [1]. *Legionella* culture in respiratory specimens is commonly used to confirm positive NAAT or urinary antigen results and is required to determine the *Legionella* species and serogroup responsible for the infection. Culture isolation also remains essential for source attribution; however, isolates can be difficult to obtain due to challenges such as (i) non-productive cough limiting appropriate respiratory specimen collection or (ii) non-viable or non-culturable bacteria or low biomass in clinical specimens [5–7]. Legionellae are also quite slow-growing, with culture isolation taking 7–10 days from both clinical and environmental specimens (Fig. 1). Characterization of isolates obtained by culture is based on whole-genome sequencing (WGS) and when combined with epidemiological data, contributes to surveillance and outbreak investigations [1, 8].

**Fig. 1.**
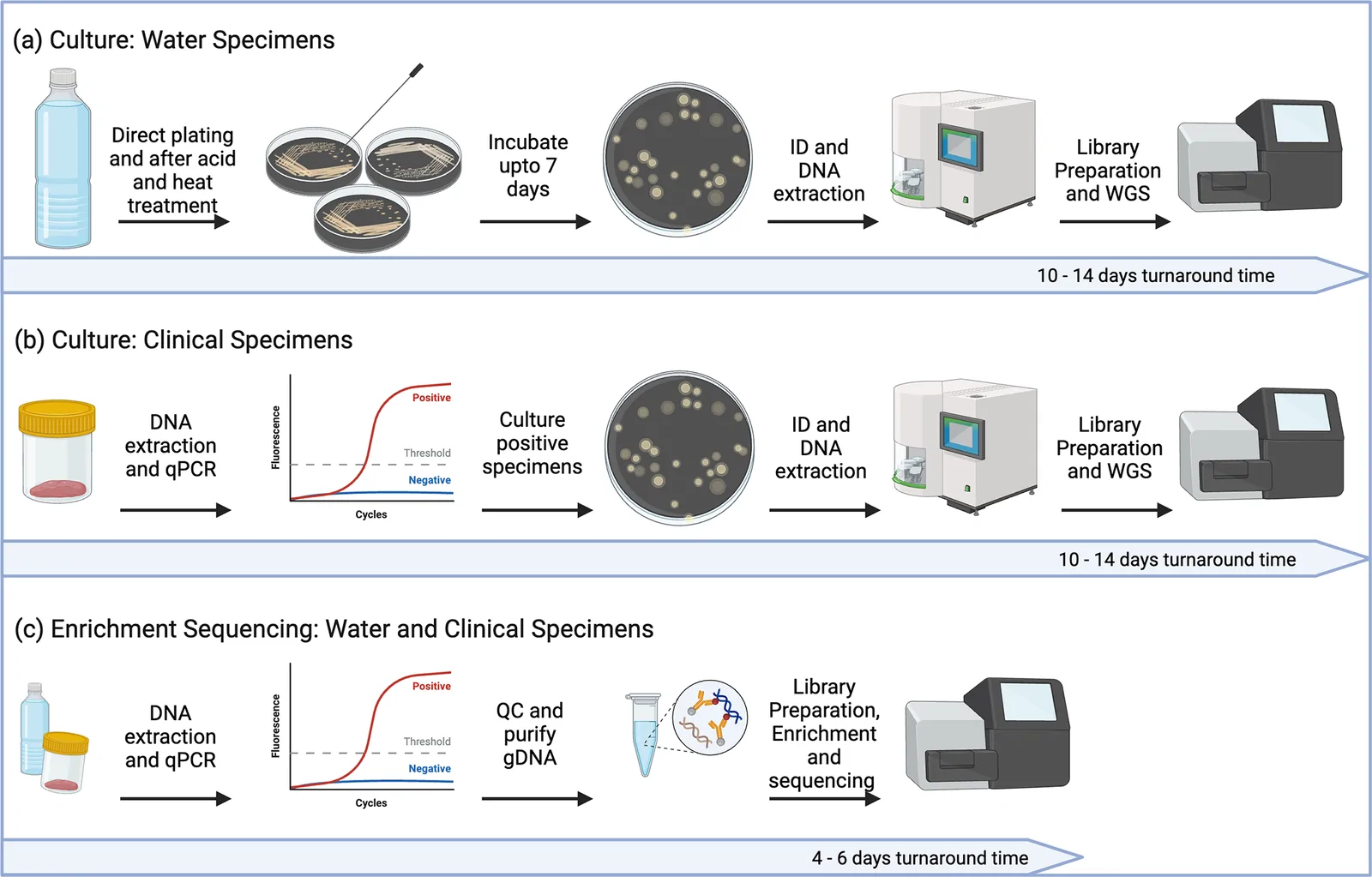
Workflow comparison for *Legionella* genome sequencing for clinical and water specimens. This schematic diagram provides an overview of current culture methods used for water (a) and clinical specimens (b), including average turnaround times to generate a sequence from a positive isolation. Traditional culture takes ~7–10 days to generate an isolate from both clinical and environmental specimens with an additional 2–4 days for WGS. In comparison, enrichment sequencing (c) bypasses the culture steps, allowing for direct sequencing from PCR-positive water and clinical specimens within 3–4 days once genomic DNA has been extracted, halving the time required to generate a sequence for analysis. Created with BioRender.com.

As sequencing technologies continue to mature and become more affordable, metagenomic sequencing is increasingly being utilized for sequencing infectious diseases in conjunction with genome enrichment methods [9–12]. This offers a targeted approach that can be used in a hospital setting for patient care or integrated into pathogen genomics workflows to improve public health responses. Metagenomic sequencing indiscriminately sequences all DNA within a specimen, whereas genome enrichment sequencing targets specific genes or pathogens within a specimen. DNA or RNA probes are used to capture prepared libraries and amplify the enriched libraries prior to sequencing. Enrichment sequencing is a cost-effective alternative to direct metagenomic sequencing as the targeted approach allows for pooling of bar-coded specimens onto a single sequencing run and enriching for pathogens of interest can provide increased sensitivity and sequencing depth for accurate characterization of pathogens. Many pathogens of interest benefit from a culture-independent approach for genome characterization, and the use of enrichment sequencing has been successfully demonstrated for surveillance of *Bordetella pertussis*, *Francisella tularensis* and *Mycobacterium ulcerans,* as well as for characterizing outbreaks of *Treponema pallidum* [9–13]. A 2024 study retrospectively applied hybridization-based enrichment sequencing to culture-negative clinical specimens, demonstrating increased recovery of *Legionella* reads when compared with pre-enriched specimens [14].

In 2024, a major *Legionella* outbreak in Melbourne, Australia, resulted in 115 confirmed cases of LD and 2 deaths [15]. The Victorian Department of Health (DoH) launched a major public health investigation, leading to the collection of 189 cooling tower specimens, with two high-risk towers identified. Laboratory investigations confirmed LpSG1 as the causative pathogen and genomic analysis of clinical and cooling tower genomes linked the outbreak to a cooling tower in Laverton in Melbourne’s west.

Here, to complement current genomic outbreak investigation practices used by public health microbiology laboratories, we employed a bacterial hybridization-based genome enrichment sequencing method targeting informative sites, exhibiting variation across a pan-*L. pneumophila* genome and demonstrated its utility in real-time during a large 2024 outbreak of LD in Melbourne, Australia [15].

## Methods

### Epidemiological methods

LD is an urgent notifiable condition under the Victorian Public Health and Wellbeing Act 2008, and a major public health investigation was initiated on Friday 26 July 2024 following a surge in notifications of legionellosis.

Epidemiological investigations were led by the Victorian DoH and supported by Local Public Health Units. Detailed case interviews were conducted to collect demographic and clinical data, and to identify potential risk factors and exposure locations. Epidemiologists analysed data on exposure sites using geospatial tools to find a possible point of origin and inform subsequent cooling tower investigations. DoH staff issued 167 notices (Victorian Public Health and Wellbeing Act 2008) to registered cooling tower operators, requiring them to provide test results and any operational changes. Staff also visited sites for testing and subsequent disinfection of cooling towers, prioritizing towers in the vicinity of the area of concern identified by epidemiological investigations.

The case definition for the outbreak was defined as ‘*L. pneumophila* 1 (LP1) cases (confirmed or probable) with onset date on or after 15th July who have part of their incubation period in Victoria.’

### Isolation of *Legionella* and whole-genome sequencing

Examination of waters for isolation of *Legionella* was performed as per AS/NZS 3896 : 2017 [16]. Briefly, water was directly inoculated onto selective agar plates using the untreated specimen and following pre-treatment with acid and heat to reduce the level of background bacteria. Agar plates were incubated and typical colonies selected for confirmation using the VITEK MS PRIME system (Biomerieux). Isolates confirmed as *L. pneumophila* were differentiated by latex agglutination (Thermo Fisher Scientific) to distinguish between LpSG1 and *L. pneumophila* serogroups 2–14 (LpSG2–14). Representative isolates identified from each species and *L. pneumophila* serogroups were retained for WGS. A total bacterial count was performed on water specimens by pour plate method as per AS/NZS 4276.3.1 : 2007 [17].

Diagnostic testing of clinical specimens was performed with urinary antigen testing at primary diagnostic laboratories, with confirmatory urinary antigen testing and subsequent NAAT and isolation of clinical isolates from respiratory specimens performed by the Victorian Infectious Diseases Reference Laboratory (VIDRL).

Genomic DNA (gDNA) was extracted from a single colony on the automated QIAsymphony platform using the QIAsymphony DSP Virus/Pathogen Mini Kit (Qiagen), as per the manufacturer’s instructions. DNA libraries were prepared for sequencing using the Nextera XT library sample preparation workflow (Illumina), and libraries were sequenced on an Illumina NextSeq 2000 instrument using 150 bp paired-end reads following instrument-specific instructions.

Raw bcl files were converted to fastq files using bcl2fastq v2.19.0.316 [18], maintaining Illumina Quality Scores (Qscore) generated by the sequencer. Quality control (QC) of reads including genome assemblies and species identification was conducted using the microbial genomics pipeline, bohra v2.3.0 [19]. The phylogeny pipeline of bohra was run using default parameters using seqkit v2.1.0 [20], *kraken2* v2.1.2 [21], KMC v3.2.1 [22], shovill v1.1.0 [23] and SPAdes v3.15.2 [24]. QC required a minimum of 40× average depth of coverage in resulting contigs and an average Qscore of >30. The resulting contigs were also processed in bohra using snippy v4.4.5 [19, 25] to find SNPs in sequencing reads and snp-dists v0.8.2 [26] to create an SNP distance matrix and infer a phylogeny for cluster detection, using the local reference genome AUSMDU00010536 (accession number CP045974.1). Details of all genomes are available in Table S1, available in the online Supplementary Material.

Isolation of *Legionella* from cooling tower specimens and subsequent sequencing and analysis of WGS data were performed using validated workflows independently accredited by the National Association of Testing Authorities, Australia.

### Enrichment sequencing probe design

A pan*-L. pneumophila* RNA bait set was designed using genome sequence diversity from a panel of 709 LpSG1 isolates derived from human and environmental sources across Victoria, Australia, supplemented with publicly available international genomes representing broad geographic diversity, including Europe, North Africa, the Middle East, East Asia, North America and Australia and temporal diversity, with isolates collected between 1986 and 2018 (Table S3). To capture accessory and variable genomic content across this panel, 120 bp sequences were enumerated from the 709 genome assemblies using kounta v0.2.3 (https://github.com/tseemann/kounta). Candidate 120-mers were filtered by minor allele frequency to enrich the bait-design input for strain-informative pan-genomic regions, retaining 120-mers present in ≤5% of the 709 assemblies before clustering at 75% nt identity using VSEARCH v2.14.2 [27], yielding 52,231 120-mers. The retained clustered sequences were assembled into 9,114 contigs using Velvet v1.2.10 [28] to reduce redundancy among overlapping sequences and provide contiguous pan-genomic target regions suitable for SureSelect probe design. The resulting pan-genome contigs were combined with the complete chromosome and plasmid sequences from a *L. pneumophila* clinical isolate originating from Melbourne, Australia (GenBank assembly accession: GCA_900092465.1) and used as the target sequence input for bait design.

Baits were designed at ~1× tiling density using the online *eArray* platform for the *SureSelect* target enrichment system (Agilent Technologies), and the final pan-genomic bait set comprised 28,061 probes (2.8 Mbp) (Table S4) and, when mapped to the *L. pneumophila* AUSMDU00010536 reference genome (3.4 Mbp), represented ~82% of the *Legionella* chromosome (Fig. S1).

To assess recovery of SNP variation across the broader LpSG1 by the final bait set, the 709 genome assemblies were randomly fragmented *in silico* into 300 bp fragments. SureSelect baits were aligned to these fragments using blastn v2.17.0 [29], and fragments were classified as captured if they aligned to at least one bait with ≥85% nt identity over ≥80 bp. Captured fragments were retained per isolate and analysed with split kmer analysis (SKA) v1.0 [30] (using default parameters of k=15 and m=0.2), and capture performance was measured as the proportion of SKA variants detected from the original 709 assemblies that were also detected from the captured fragment datasets.

### Preparation of simulated water specimens

Simulated environmental specimens were prepared to evaluate the performance of the enrichment sequencing assay using the pan-genome *Legionella* bait set. A negative background for simulated specimens was generated by pooling previously tested culture-negative cooling tower water specimens. Pooled water specimens were confirmed as *L. pneumophila* negative using quantitative PCR (qPCR) as described below. Simulated positive specimens were then prepared by aliquoting into 99 ml volumes, retaining one as a negative control (Fig. S2). A single colony of *L. pneumophila* type strain (ATCC 33152) was inoculated in 1,800 µl of PBS and serially diluted to 10^−6^ before plating on buffered charcoal yeast extract (BCYE) agar plates for enumeration. Enumerated *L. pneumophila* in PBS were combined with the aliquoted negative background water specimens (1 ml in 99 ml) to achieve c.f.u. ml^-1^ values of ~10, 50, 100, 500 and 1,000 c.f.u. ml^−1^. A 100 ml volume of simulated specimens was vortexed briefly before being processed through vacuum-based filtration, using 47 mm diameter, 0.45 µm pore size, nitrocellulose membranes (Millipore) in preparation for DNA extraction (Fig. S2). Specimens were assessed by qPCR to establish Cq values at the increasing microbial loads.

### Genomic DNA extraction

Extraction of respiratory specimens was performed at VIDRL. Respiratory specimens were treated with Sputasol (Thermo Fisher Scientific) followed by 10 min of incubation at 37 °C. Specimens were then centrifuged at 12,000 ***g*** for 5 min, and the supernatant was removed leaving 200 µl of treated sample which was extracted on the Freedom EVO 100 platform (Tecan) using the Quick DNA/RNA™ MagBead Extraction kit (Zymo Research) as per the manufacturer’s instructions. A host DNA depletion step was not performed prior to extraction of clinical specimens.

Both simulated and outbreak water specimens were concentrated by filtering 100 ml through a 47 mm, 0.45 µm filter membrane. The filter membrane was transferred to 10 ml PBS and was manually broken down with scissors (sterilized by alcohol and flame). The membrane was vortexed for 2 min and incubated for 10–15 min at room temperature before 400 µl of the resuspension was transferred to a sterile microcentrifuge tube for DNA extraction. gDNA was extracted from the 400 µl suspension using the EZ2 automated platform and the Virus Mini Kit v2.0 (Qiagen), as per the manufacturer’s instructions, with a final elution volume of 60 µl.

### Quantitative PCR

Recovered *L. pneumophila* concentrated gDNA was assessed by qPCR using primers and probes as described in ISO/TS 12869 [31]. Multiplex qPCR was run on a CFX96 Touch Real-Time PCR Detection System (Bio-Rad Laboratories) for 2 min at 50 °C, 10 min at 95 °C, and 45 cycles consisting of 15 s at 95 °C and 1 min at 60 °C. Each 25 µl reaction contained 2.5 µl of gDNA, 12.5 µl SensiFAST Probe No-ROX Mix (Meridian Bioscience), 200 nM of each of the forward, reverse primers and probes for *L. pneumophila* and *Legionella* 16S rRNA gene and 1× qPCR Extraction Control Red (cat # MER-MDX026-10, Meridian Bioscience).

### Genome enrichment sequencing of outbreak specimens

Genome enrichment by hybridization capture was performed using the Illumina DNA Prep with Enrichment kit (Catalogue # 20025524) using Agilent SureSelect RNA baits to enrich for *L. pneumophila* as described above. gDNA extracted from clinical and environmental specimens was initially concentrated using AMPure XP beads (Agilent), followed by enzymatic DNA fragmentation with simultaneous end repair and A-tailing. The tagmented DNA was amplified and index adaptors were ligated. A maximum of 12 libraries were pooled in a single hybridization reaction. Hybridized probes were captured and the enriched library was amplified using the PCR primer cocktail provided with the enrichment kit. After a final clean-up, the index library pool was quantified using fluorimetry (Qubit dsDNA high sensitivity assay, Thermo Fisher) and its quality was assessed by electrophoresis using the High Sensitivity D1000 ScreenTape run on the TapeStation (Agilent). The resulting libraries were sequenced on an Illumina NextSeq 2000 using paired-end 150 bp chemistry.

### DNA sequence read processing

Sequence reads were processed with fastp v0.23.4 [32] to remove adapters, and kraken2 v2.1.2 [21] with the plusPF database (6 May 2024) was used to determine the percentage of each read set assigned to *Legionella* species and to quantify off-target sequencing, such as human DNA in clinical specimens or other microbial DNA in water specimens. The reads were aligned to the *L. pneumophila* Philadelphia 1 strain complete genome AE017354 [33] using minimap2 v2.24 [34], and unaligned reads were discarded. Kraken2 was rerun on the filtered reads to confirm that only *Legionella* reads were present, and genome metrics were assessed with seqkit stats [20]. Enriched *Legionella* reads were mapped to the reference genome AUSMDU00010536 to evaluate coverage and mean depth (Table S2). A >70% coverage threshold was empirically chosen as the cut-off for read sets to be included in subsequent core genome phylogenetic analyses. Enriched read sets with >70% coverage all had an average read depth above 180×. These were subsampled to ~150× before SNPs were called with snippy [25] using the reference genome AUSMDU00010536.

### Phylogenomic analysis

Phylogenies were inferred using a maximum-likelihood method in IQ*-*TREE *2* v2.1.4-beta [35] with the GTR+f+G4 model, based on core-genome alignment of enriched reads together with draft genomes from outbreak isolates and the library of Victorian *L. pneumophila* genomes by running the SNP pipeline of bohra using local reference strain AUSMDU00010536 [19]. The analysis was repeated on a subset of genomes encompassing the outbreak clade with draft genome from index case AUSMDU00105903 for the reference to generate an SNP distance matrix with greater core genome coverage for cluster analysis.

### Comparative genomics

To support the core genome phylogenetic analysis, an alternative reference-free SNP alignment was generated using SKA from the Illumina reads using default parameters (k-mer length of 15 and minor allele frequency of 0.2) as well as parameters optimized for bacterial surveillance [parameters: k-mer length (k) of 19 and minor allele frequency (m) of 0.01] [36]. SKA produced a pairwise distance matrix, from which genomic clusters were generated in R using the ggraph, tidygraph and igraph packages. Clusters defined by a Jaccard index of ≥0.99 and SNPs≤5 were chosen for closer investigation.

In addition, a machine-learning model was used for source-attribution classification. SKA was run using the same parameters as the previously published LpSG1 source-attribution framework: a k-mer length of 15, minor allele frequency of 0.2 and a minimum alignment split k-mer prevalence of 0.1. The resulting SNP matrix was one-hot encoded before training the multivariable classifier, as previously described [37].

## Results

### Validation of genome enrichment sequencing using hybridization capture

Genome enrichment sequencing using hybridization capture with the novel RNA bait set was validated using simulated cooling tower water specimens spiked with *L. pneumophila* ranging between 10 and 1,000 c.f.u. ml^−1^ in 100 ml volumes. The presence of *L. pneumophila* in the resulting gDNA was measured by qPCR in triplicate before specimens were subjected to enrichment sequencing. In parallel, direct genome sequencing was performed on the same gDNA extracts obtained from the simulated water specimens to compare the impact of enrichment on subsequent sequence coverage and variant calling.

The simulated specimens provided a theoretical limit of detection (LOD) for environmental specimens. Culture of *Legionella* using AS/NZS 3896 : 2017 has an LOD of 1.0 c.f.u. ml^−1^, and therefore, a water specimen with a volume of 100–1,000 ml would be expected to contain up to 1,000 *L*. *pneumophila* cells [16]. If complete recovery was achieved through filtration and gDNA extraction, the maximum yield would be 1,000 genome copies giving a predicted quantification cycle (Cq) value of ~34. This was observed in simulated specimens (Fig. S2), where gDNA was extracted from specimens containing 10 c.f.u. ml^−1^ and shown to have Cq thresholds of ~34, while comparable gDNA prepared from specimens containing 50–500 c.f.u. ml^−1^ were shown to have Cq thresholds of 28–31.

Following sequencing, we observed fewer total reads in enriched read sets compared to direct sequencing; however, the percentage of reads mapping to the *L. pneumophila* AUSMDU00010536 chromosome was higher when compared to reads from direct genome sequencing from the simulated water specimens. This pattern was seen across all bacterial input doses (Fig. S3). For instance, at 10 c.f.u. ml^−1^ (Cq=34.1), 75% of reads mapped to the *Legionella* reference genome, achieving coverage of 49% of the genome while direct sequencing had only coverage of 21% of the genome with <1% of reads mapping to the *Legionella* reference genome (Fig. S3 and Table S5). At the higher bacterial loads, near complete genome coverage (99.9%) was observed in direct sequencing reads at 500 and 1,000 c.f.u. ml^−1^ (Cq=28.8 and 27.9, respectively), while only 33% of reads mapped to the *Legionella* reference chromosome, increasing to 69% at 1,000 c.f.u. ml^−1^. In contrast, near-complete genome coverage was observed for the probe set (2.8 Mbp) in enriched sequencing reads at 500 and 1,000 c.f.u. ml^−1^ (83 and 85% of the complete genome), with >97% of reads mapping to the *Legionella* reference genome (Fig. S3 and Table S5). These experiments show a clear logarithmic increase in *Legionella* reads and coverage of the AUSMDU00010536 chromosome when using enrichment sequencing (Fig. S3).

To assess whether the bait set was expected to recover variation across the broader LpSG1 population, we performed an *in silico* capture simulation using the 709 assemblies used for bait design. Through 300 bp fragment simulations, 88.73% of SKA variants identified from the original assemblies were also detected in the captured-fragment datasets (173,264 and 153,744 SNPs detected among the 709 original assemblies and captured fragments, respectively), indicating that the bait set had the capacity to recover substantial SNP diversity across the LpSG1 collection.

### Epidemiological investigation of the 2024 LD outbreak

Health authorities in the major metropolitan city of Melbourne (population 5.3 million in 2024) in southeastern Australia were alerted to a significant increase of LD in late July 2024. The first cases were notified on Friday 26 July 2024 and case numbers increased rapidly with the outbreak concluded on the 20th of August. There were 115 confirmed cases in total with an illness onset date between 19 and 26th July for most cases. Of the 115 cases, 110 were hospitalized and there were 2 fatalities. Cases presented with typical symptoms of fever, chills, cough, headache and muscle aches and pains. A total of 15 LpSG1 isolates were obtained from 13 patients as part of the outbreak investigation (Table S1). A comprehensive description of the epidemiological investigation will be reported in a forthcoming manuscript.

The cases were spread over a large geographic area of metropolitan Melbourne, with the initial epidemiological investigation finding multiple potential exposure sites concentrated in Melbourne’s west. This led health authorities to investigate 70 sites, collecting 189 water specimens from 56 cooling towers for microbiological assessment. All cooling tower specimens were submitted to the Microbiological Diagnostic Unit Public Health Laboratory (MDU PHL) for testing.

### Clinical microbiology

A total of 203 respiratory specimens were submitted to VIDRL for *Legionella* testing by PCR. PCR detected *L. pneumophila* in 23 respiratory specimens, and although PCR does not distinguish serogroup, each case had a corresponding positive LpSG1 urinary antigen result. In total, 13 respiratory specimens were also positive by culture, in addition to their positive urinary antigen and PCR results from the same patient.

### Cooling tower screening to support the LD outbreak investigation

A total of 189 cooling tower waters were cultured for the presence of *Legionella* species as per AS/NZS 3896 : 2017. Five positive specimens were identified from two separate cooling towers (CT-1 and CT-2). Both cooling towers were confirmed as LpSG1 positive with no other *Legionella* species detected. *Legionella* enumeration results for CT-1 ranged from 500 to 200 c.f.u. ml^−1^ in positive specimens with subsequent water collections from CT-1 all confirming the cooling tower had a negative *Legionella* result (Table S6). CT-2 had three positive LpSG1 specimens with counts ranging from 20 to 200 c.f.u. ml^−1^. A total of 51 colonies were selected from CT-1 and CT-2 for WGS (Table S1). WGS results showed that the LpSG1 colonies were indistinguishable by genomic analysis within each of the two cooling towers.

qPCR was used to rapidly screen 56 randomly selected cooling tower specimens for *Legionella* and *L. pneumophila*. Of the 56 specimens, 59% (*n*=33) were PCR-positive for *Legionella* spp. of which 5 (9%) were PCR-positive for *L. pneumophila* with specimens yielding Cq values between 32 and 37. Specimens with a Cq>38 were considered as negative as this is above the LOD for culture and enrichment sequencing as demonstrated with simulated specimens. All *L. pneumophila* PCR-positive specimens were LpSG1 culture-positive and had total bacterial counts of <2,000 c.f.u. ml^−1^ (Table S6). The 28 specimens that were PCR-positive for *Legionella* spp. only were all culture-negative with this discrepant result likely explained by the presence of non-culturable bacteria or free *Legionella* DNA in those specimens, as has been previously reported [38]. Internal control cycle thresholds group around cycle 30, with the manufacturer reporting an expected cycle threshold of 29±1.

### Comparative LpSG1 genomics to inform the outbreak response

A total of 51 LpSG1 colonies were cultured from the 5 PCR-positive water specimens obtained from 2 cooling towers (CT-1 and CT-2), and together with the 15 clinical LpSG1 cultures, they were all subjected to WGS. All case and environmental isolations were confirmed as LpSG1. A total of 23 specimens (*n*=5 water and *n*=18 clinical specimens out of a possible 23) were sequenced using the hybridization capture method during the outbreak investigation. Clinical specimens with a low Cq or where clinical specimens were culture negative were preferentially selected for enrichment sequencing. The resulting sequencing reads were analysed together with a historical local collection of 592 publicly available *L. pneumophila* genomes, obtained from 196 clinical and 395 environmental specimens (Supplementary Fig. S4).

To validate results generated from the phylogenetic analysis, SKA [30] was used as a secondary independent pairwise comparison method as it captures variation across all shared loci and avoids reference bias, thereby incorporating both core and accessory genome SNPs; an approach valuable for distinguishing outbreak from non-outbreak isolates.

Both the phylogenetic and SKA-based analysis showed concordant results (Table S7). All the outbreak patient isolates and the 32 environmental isolates from CT-1 formed a single outbreak clade. Two historical Victorian cases from 2015 and 2023 (AUSMDU00019457 and AUSMDU00082839) were highly related to the outbreak strains based on the genomic analysis, as can be seen in yellow in Fig. 2, with only one SNP distinguishing both from outbreak isolates. However, both cases were singletons with no epidemiological link to the outbreak established. The core alignment contained 206,179 SNPs of which 180,971 were parsimony informative sites. The outbreak clade was distinct from all other isolates in the analysis with >300 unique SNPs separating it from the nearest clade. The 19 environmental isolates sequenced from CT-2 were genomically distinct from the outbreak clade and therefore excluded as a potential source of the outbreak. WGS of case and environmental isolates confirmed the source of the outbreak to a single cooling tower in Melbourne’s west. CT-1 was subsequently disinfected, and the outbreak quickly controlled.

**Fig. 2.**
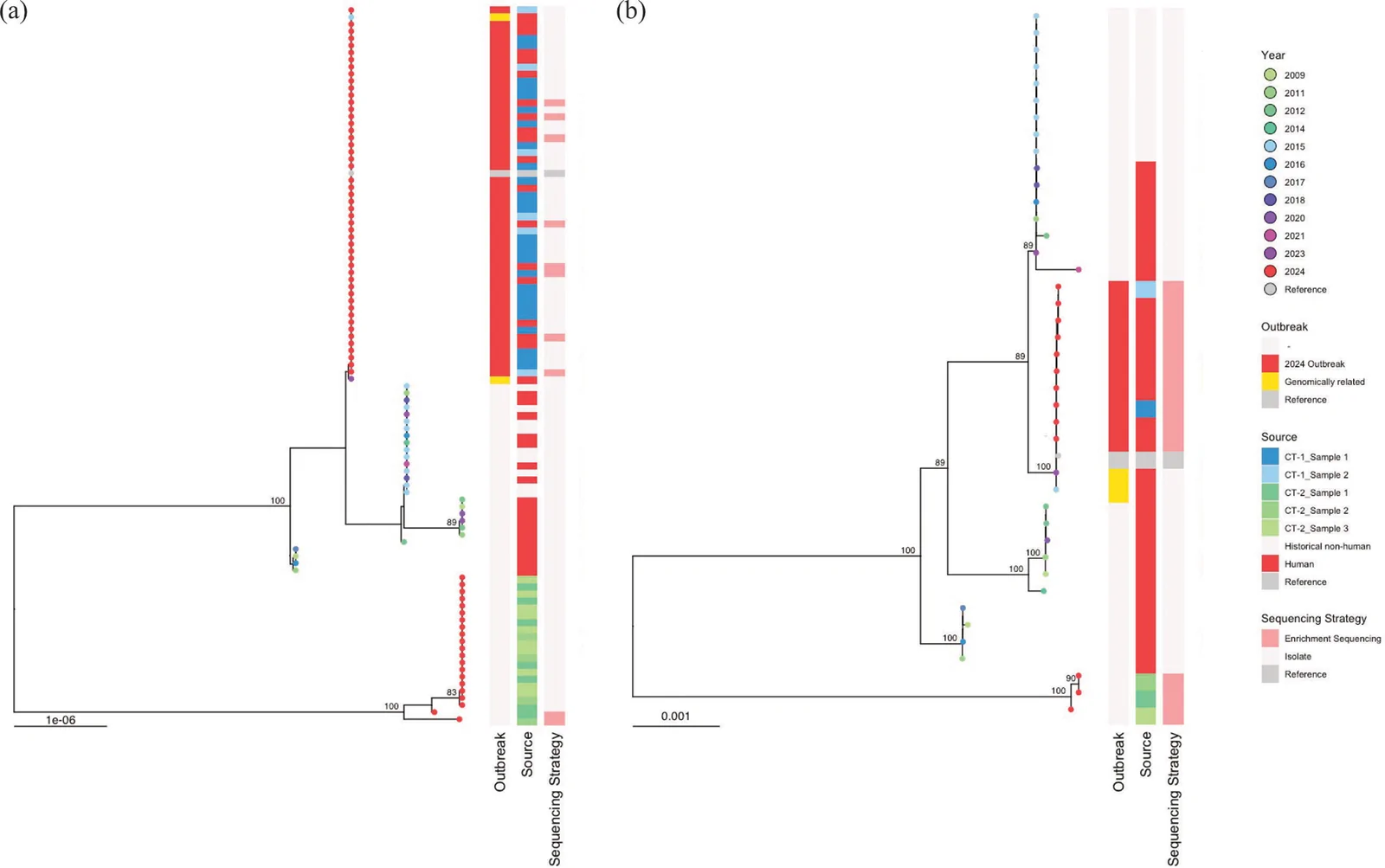
Maximum-likelihood phylogeny of clinical and environmental *L. pneumophila* genomes collected as part of the 2024 outbreak. All phylogenies were constructed with IQ-TREE 2 [35] and visualized in R v4.3.1 using the ggtree v3.12.0, treeio v1.28.0, ggplot2 v3.5.2, readr v2.1.5 and ape v5.8–1 packages. (a) Analysis of a subset of genomes from the initial full analysis encompassing the outbreak clade and including enrichment sequencing reads. The draft genome from index case AUSMDU00105903 was used for the reference to generate an SNP distance matrix with greater core genome coverage for cluster analysis. Enrichment sequencing reads clustered with their respective isolates in the analysis. (b) Re-analysis of the subset tree, omitting outbreak isolates, generated the same outbreak clade with enrichment sequencing read sets only.

Enriched sequencing data was analysed simultaneously with WGS data from isolates. Enriched read sets from both clinical and environmental specimens displayed variations in coverage, depth and resolution when compared to isolates (Figs 3 and S5). Clinical specimens were affected by the presence of host reads, which reduced the proportion of usable bacterial sequences. As the proportion of *Legionella* reads in a clinical specimen decreased, so too did the mean depth and coverage of the genome, resulting in genome coverage dropping below 70% at Cq=31. In contrast, environmental specimens performed slightly better, maintaining higher coverage even in cases where the Cq exceeded 32. The negligible background contamination in these specimens enabled a greater proportion of reads to be classified as *Legionella*, enhancing their analytical utility. However, for all specimens, coverage dropped when the Cq value exceeded 34. Enriched read sets displayed regions of both increased and decreased coverage across all specimens, independent of probe density across the LpSG1 reference genome (Fig. S5). Elevated coverage was associated with regions of high G+C content, whereas coverage gaps were consistent with true sequence divergence between outbreak isolates and the reference genome AUSMDU00010536.

**Fig. 3.**
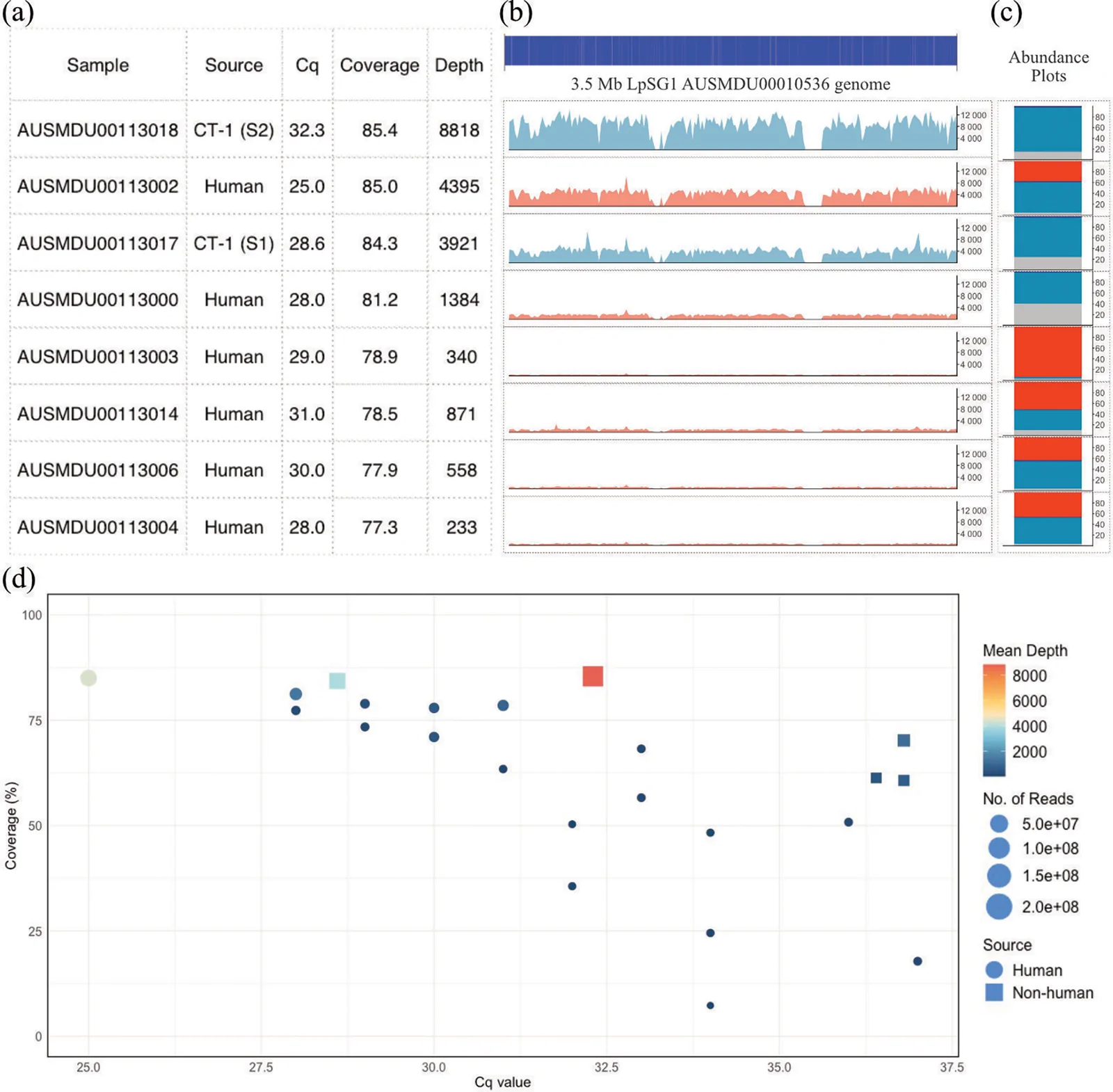
Genome enrichment sequencing of outbreak specimens. (a) Summary metrics of top-performing human and cooling tower specimens. (b) Coverage plots illustrating enriched reads of cooling tower specimens (blue) and clinical specimens (red) mapped to the *L. pneumophila* serogroup 1 reference genome AUSMDU00010536. The blue plot across the top depicts probe coverage across the LpSG1 genome, and the y-axis in each specimen plot gives the number of mapped reads across the genome. (c) Abundance plots show the percentage of *Legionella* reads (dark blue) and *L. pneumophila* reads (light blue) with respect to human (red) and other non-human (grey). (d) Coverage of the LpSG1 reference genome AUSMDU00010536 expressed as a percentage for each specimen, with specimen qPCR Cq values shown on the x-axis. The number of reads classified as *Legionella* is depicted by the size of the data point, and the average mean depth is represented by the colour of the data point.

Genome information generated through enrichment sequencing was able to reaffirm conclusions drawn from cultured isolates. Sequences obtained through this method clustered closely with their respective outbreak isolates. Additionally, when enriched data from environmental specimens was analysed with outbreak isolates, CT-1 specimens joined the outbreak clade, while CT-2 correctly diverged from the outbreak clade, reinforcing conclusions drawn from the original phylogenetic analysis. The analysis was also repeated without any culture isolate genomes from outbreak cases or water specimens. Here, the same outbreak clade was recapitulated, with cases clustering with CT-1 specimens and the two historical cases from 2015 and 2023. CT-2 specimens were again genomically distinct from the outbreak clade (Fig. 2). This indicates that hybrid capture was effective in enriching target sequences without introducing bias in phylogenetic placement.

### Machine learning accurately identified outbreak-related clinical isolates

A recently developed machine learning framework for *L. pneumophila* source attribution [37] was applied to estimate the probability that 15 clinical isolates and 18 sequence capture-enriched clinical specimens originated from the suspected cooling tower source. Model training was performed on a set of 65 environmental isolates (34 outbreak related and 31 non-outbreak related) (Table S8), and model selection was undertaken through ten repeated 80 : 20 training-validation splits of the training dataset. Combinations of feature number, classifier kernel and class weighting were evaluated using the F1-score. Optimal performance on the environmental training dataset was obtained using all 143,288 one-hot encoded features with a support vector classifier employing a sigmoid kernel (a model that maps feature relationships into a squashed S-shaped function, allowing for non-linear class separation) and class weights of 0.5 and 1 for the non-outbreak and outbreak-associated classes, respectively. Details of all model parameters are available in Table S9.

The final model was trained using all available 60 cooling tower isolates and 5 sequence capture enrichment datasets and applied to an independent test set comprising 33 outbreak-associated datasets (15 culture-derived clinical isolates and 18 sequence capture-enriched clinical specimens) and 20 non-outbreak clinical isolates. All 34 outbreak-associated datasets occupied the highest-ranked positions, with predicted source probabilities ranging from 0.984 to 1.000, where probabilities closer to 1 (on a 0–1 scale) indicate stronger association with the outbreak genotype. The next highest-ranked datasets were two non-outbreak isolates, AUSMDU00082839 and AUSMDU00019457, with probabilities of 0.983 and 0.976, respectively. The remaining 18 non-outbreak isolates had substantially lower probabilities, ranging from 0.681 to 0.014. Unrelated cases were effectively separated by their lower rankings, except for two non-outbreak clinical isolates that were highly genomically related yet were still assigned probabilities lower than all 33 outbreak-associated isolates (Table S10).

## Discussion

In this study, we developed and demonstrated the utility of a pan-genome enrichment sequencing assay for *Legionella* clinical and environmental specimens in real-time during a city-wide outbreak of LpSG1 using Agilent SureSelect target enrichment technology RNA baits. The enrichment method targets strain-informative pan-genomic regions across the LpSG1 population to maximize recovery of genomic variation relevant for source attribution. *Legionella* outbreaks remain a public health risk with man-made water systems often reported as the source despite many countries putting in place guidelines and regulations to both control and monitor *Legionella* in water systems [39–43]. In the state of Victoria, Australia, legionellosis is an urgent notifiable condition, and cooling towers must be maintained and tested as outlined in the Victorian Public Health and Wellbeing Regulations 2019. Cooling tower specimens collected for routine screening and as part of any investigation by the Victorian DoH are submitted to the MDU PHL for detection and isolation of *Legionella* spp., and all clinical isolates are submitted to MDU PHL for WGS. Routine surveillance has created a rich database of *Legionella* genomes, providing a strong genomic context for Legionnaires’ outbreak surveillance.

### The role of enrichment sequencing in outbreak investigations

The application of enrichment sequencing in *Legionella* outbreak investigations represents a significant advancement in the utility of genomics for public health and clinical microbiology. Traditional methods, such as culture-based isolation and WGS of isolates, remain the gold standard for outbreak tracking and source attribution, but they have inherent limitations. Culture is slow, requiring 7–10 days, and fails where *Legionella* is present at low abundance or in a non-viable state in clinical specimens. PCR-based screening can provide presence/absence information but lacks the resolution needed for strain-level discrimination. Additionally, adoption of qPCR for screening water specimens for the presence of *Legionella* remains challenging due to the detection of DNA outside of viable cells, translating to a high number of false positives, as has been previously reported and demonstrated in this study [38, 44]. Enrichment sequencing allows for rapid genomic characterization even in culture-negative specimens (Fig. 1). This is particularly valuable when working with primary respiratory specimens, which often contain high levels of host DNA, and environmental water specimens, where background microbial noise can obscure *Legionella* detection. The utility of enrichment sequencing has been successfully demonstrated in surveillance programmes of several pathogens, including *Legionella* in human and animal biological specimens [11, 12, 14, 45]. To our knowledge, this study is the first to demonstrate its utility in a public health investigation capturing biological and environmental specimens.

The reagent and consumable cost of hybridization-based enrichment sequencing is ~7× higher when compared to traditional culture and WGS ($800 and $110 AUD, respectively, in this study), with the latter costing including one genome per specimen only. A formal cost-benefit analysis of rapid turnaround times is yet to be assessed for enrichment sequencing; however, early genomic insights that can inform outbreak containment measures or targeted epidemiological interventions can enable faster control of transmission and reduce the overall disease burden, making it invaluable in public health investigations.

An important consideration in the application of enrichment sequencing, particularly for environmental specimens, is the potential for mixed *Legionella* populations within a single specimen. Culture-based isolation allows for WGS of individual colonies. Hybridization capture has the potential to enrich all *Legionella* DNA present in a specimen, meaning multiple strains may be present in *Legionella* reads. While mixed *Legionella* populations were not a factor in the 2024 outbreak investigated here, they have been reported in clinical and environmental water systems, where multiple strains can co-exist [46, 47]. The bait set designed in this study was specific for LpSG1; however, this does not impede possible hybridization if a non-*L. pneumophila* strain were present, and short-read sequencing data has limitations in resolving mixed populations [48]. Read lengths are insufficient to produce accurate strain-level assemblies. Evidence of a mixed population can be identified through several indicators, including the presence of variants at intermediate allele frequencies (0.1–0.9), uneven genomic coverage and fragmented assemblies with higher-than-expected contig counts. In an outbreak context, we recommend applying within-sample allele frequency analysis as a routine QC step and treating genomic typing results with caution where mixed populations are identified. Long-read sequencing platforms, such as Oxford Nanopore Technology, may offer improved strain-level resolution in these scenarios and warrant further investigation for application in *Legionella* outbreak genomics [49].

### Sensitivity and genome recovery

Our findings highlight that enrichment sequencing enables satisfactory genome recovery for phylogenetic analysis, even at low bacterial loads. Enrichment sequencing achieved near-complete coverage across the probe set (85% of the reference genome) in a simulated specimen with a LpSG1 bacterial load of 100 c.f.u. ml^−1^. Even at 10 c.f.u. ml^−1^, coverage remained at ~50%, while direct sequencing at this concentration dropped to ~20% coverage in simulated specimens. This increased sensitivity makes enrichment sequencing particularly useful in environmental monitoring, where *Legionella* may be present at low levels but still pose a public health risk. *In silico* capture simulation further indicated that the bait set retained broad population-level applicability, recovering >88% of genomic variants across 709 LpSG1 assemblies.

Results observed in outbreak specimens demonstrate that the success of enrichment sequencing is dependent not only on the bacterial load in the primary specimens, but also on the specificity to the target organism. This was evident when comparing results observed in the clinical DNA extracts versus environmental specimens (Fig. 3). Clinical specimens exhibited high coverage across the *Legionella* genome (71–85%) when specimens had a Cq of <31, with coverage dropping as Cq values increased. In contrast, 85% coverage was achieved in environmental specimens with a Cq of 32 and maintained at 60–70% in specimens with a Cq of 36–37. These high Cq environmental specimens had a large proportion of reads mapping to *Legionella* and a mean depth of 500–1,000×. In clinical specimens, however, target genome coverage appears to be somewhat compromised due to a higher degree of host DNA background and carryover, where ~50% or fewer of reads are classified as *L. pneumophila*. Re-design of the probe set would be required to increase specificity, or a host DNA depletion step may be included prior to extraction to improve resolution in clinical respiratory specimens.

### Integration with standard genomic pipelines

A key advantage of enrichment sequencing is its seamless integration with existing validated and accredited WGS outbreak analysis pipelines. Standardized pipelines capable of characterizing pathogens and running a phylogenetic analysis, such as borha, are routinely applied following genome QC to characterize pathogens and perform phylogenetic analyses [19, 50, 51]. Genome QC typically includes assessment of parameters such as average sequencing depth, number of contigs and confirmation that the genome is classified as the expected organism. Similar principles can be applied to enrichment sequencing data, with thresholds appropriately optimized for this approach. In this study, poor-quality data reduced the proportion of the core genome that could be confidently analysed; therefore, only specimens with >70% reference genome coverage were included in downstream analyses. Further validation is required to establish optimal QC thresholds.

Additional factors, including the choice of reference genome and the genetic diversity of specimens, can also influence phylogenetic analyses and their interpretation [52, 53]. Ideally, a high-quality reference genome closely related to the outbreak clade should be used; however, when unavailable, a draft *de novo* assembly closely related to the outbreak lineage is a suitable alternative [54]. *L. pneumophila* has been described as a clonal organism with large genetic distances reported between unrelated genotypes (>36,000 SNPs) whilst the same genotype can persist over large temporal ranges [55, 56].

Importantly, enriched genomes clustered accurately with their corresponding outbreak isolates, confirming that the method does not introduce bias into phylogenetic reconstructions of *L. pneumophila*. Consistent phylogenetic relationships were observed both when enrichment sequencing data were analysed independently and when combined with traditional isolate-based WGS data from the outbreak. This demonstrates that enrichment sequencing can operate as both a standalone approach and a complementary tool within existing established genomic surveillance pipelines.

In parallel, SKA was employed to generate a reference-independent SNP alignment for machine learning classification. This approach avoids reference bias by capturing variation across all shared loci, ensuring both core and accessory genome SNPs contribute to the analysis. Importantly, the machine learning model produced findings concordant with the phylogenomic approach: both methods consistently separated all outbreak-associated datasets from unrelated clinical isolates, while both also highlighted the difficulty of distinguishing two non-outbreak clinical isolates that were genomically highly similar to the outbreak cluster yet had no epidemiological link (AUSMDU00082839 and AUSMDU00019457). These results demonstrate that enrichment sequencing data can be seamlessly integrated into both read-mapping-based phylogenomic analyses and SKA-based machine learning pipelines, with both approaches providing complementary and mutually reinforcing evidence for accurate outbreak delineation.

### Public health implications and future directions

Hybridization-based enrichment sequencing represents a major step forward in *Legionella* outbreak investigations by addressing key limitations of culture-based and standard metagenomic approaches. Its ability to recover informative genomic data from non-culturable specimens enhances outbreak resolution and enables more comprehensive epidemiological assessments.

Assay enhancements may include optimizing the bait design to improve genome-wide capture efficiency and increase specificity, inclusion of a host DNA depletion step prior to extraction to improve resolution in clinical respiratory specimens and using tailored informatics and quality control (QC) systems in the workflow [57]. Addition of a process control would allow for a robust QC process for enrichment sequencing read sets. Inclusion of a process control would allow coverage and depth thresholds to be defined to ensure the minimum desired core genome would be captured in a phylogenetic analysis. Further to this, QC of the data is the missing piece required for public health laboratories to gain accreditation for enrichment sequencing workflows and analysis pipelines.

In conclusion, enrichment sequencing is a powerful complementary tool that enhances existing WGS-based outbreak investigations by improving sensitivity, accelerating response times and providing genomic resolution in challenging specimen types. By integrating this approach into routine *Legionella* surveillance, public health authorities can strengthen outbreak preparedness and response, ultimately reducing the public health burden of LD.

## Supplementary material

10.1099/mgen.0.001836 Supplementary Material 1.

10.1099/mgen.0.001836 Table S1.

## Abbreviations

Cq: quantification cycle
DoH: Department of Health
gDNA: genomic DNA
LD: Legionnaires' disease
LOD: limit of detection
LpSG1: *Legionella pneumophila* serogroup 1
MDU PHL: Microbiological Diagnostic Unit Public Health Laboratory
NAAT: nucleic acid amplification test
QC: quality control
qPCR: quantitative polymerase chain reaction
SKA: split kmer analysis
VIDRL: Victorian Infectious Diseases Reference Laboratory
WGS: whole-genome sequencing
