# Supplementary material for: Pathogen genome enrichment sequencing to support a city-wide Legionnaires’ disease outbreak investigation
Source: Microb Genom. 2026 Sep 28;12(9):001836. doi: 10.1099/mgen.0.001836 (PMC13618814; doi:10.1099/mgen.0.001836)
Supplement: Supplementary file 1 [file mgen-12-01836-s001.pdf]

Supplementary Data

Pathogen genome enrichment sequencing to support a city-wide Legionnaires' disease outbreak investigation

Mercoulia *et al.*

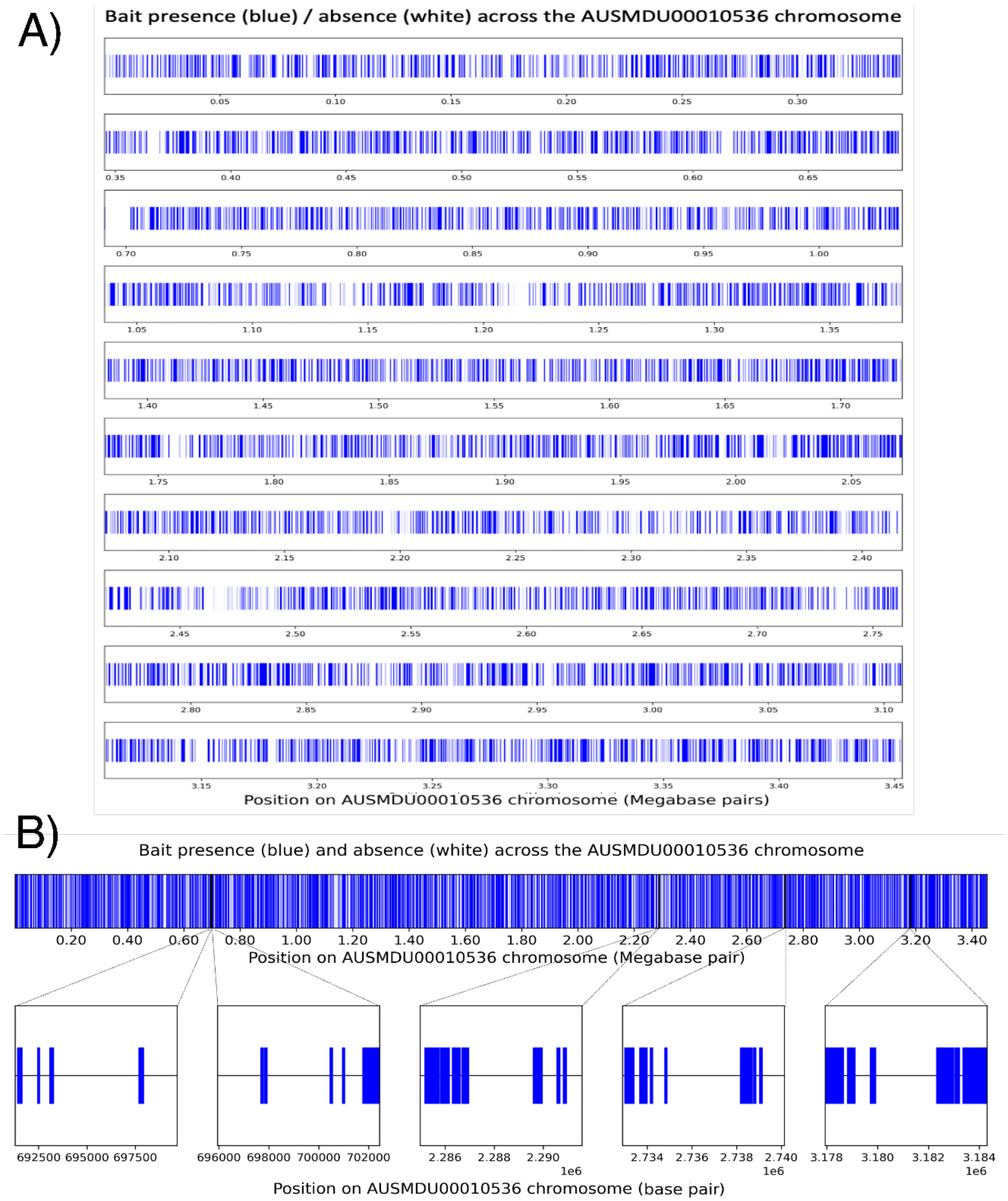

Supplementary Figure 1. Chromosome-wide distribution of bait presence and absence. (A) Zoomed-in view of consecutive segments of the AUSMDU00010536 chromosome. Each horizontal panel represents a consecutive segment of the AUSMDU00010536 chromosome. Blue vertical lines indicate positions where baits were

present, while white lines denote absence. (B) Chromosome-wide overview of bait presence (blue) and absence (white), with the genome binned into 10 bp segments for visual clarity. Top panel shows the full AUSMDU00010536 chromosome, and the five subpanels display the largest regions of continuous bait dropout. Blue ticks indicating bases where baits were present.

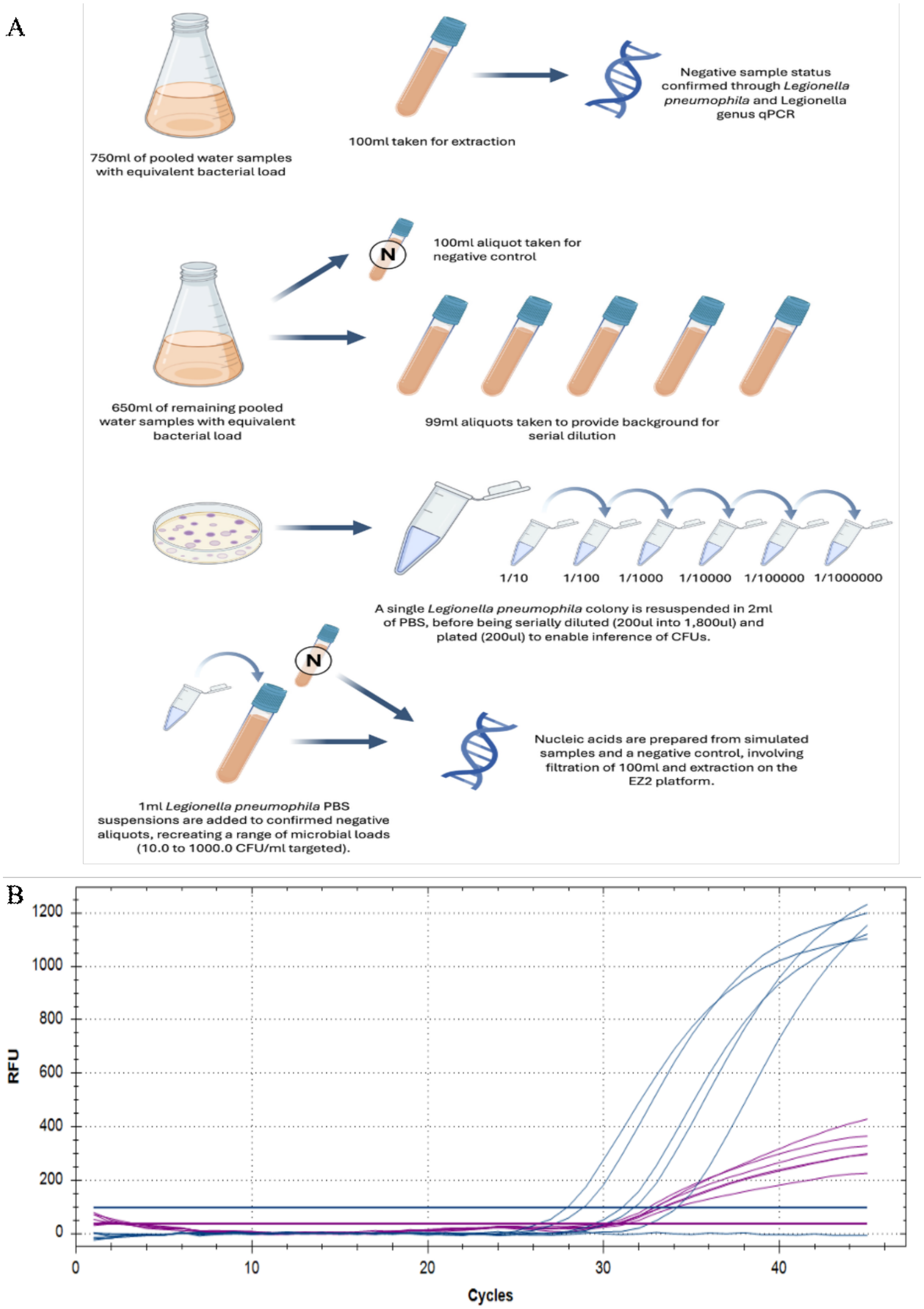

C

Microbial Load (CFU/ml)	1000	500	100	50	10	0
Cycle threshold	27.88	28.80	31.03	31.69	34.14	-
Simulated genome copies	100,000	50,000	10,000	5,000	1,000	Negative

Supplementary Figure 2. Preparation of validation samples and spiking volumes. (A) Schematic diagram outlining steps taken to prepare simulated samples used to validate enrichment sequencing method. (B) Amplification curves for *Legionella pneumophila* qPCR (blue, FAM) and internal Bioline controls (red, CY5) and (C) corresponding microbial loads and Cq values for *L. pneumophila* amplification curves. Genomic DNA extracted from 1000 CFU of Legionella pneumophila are shown to have a cycle threshold of ~34.

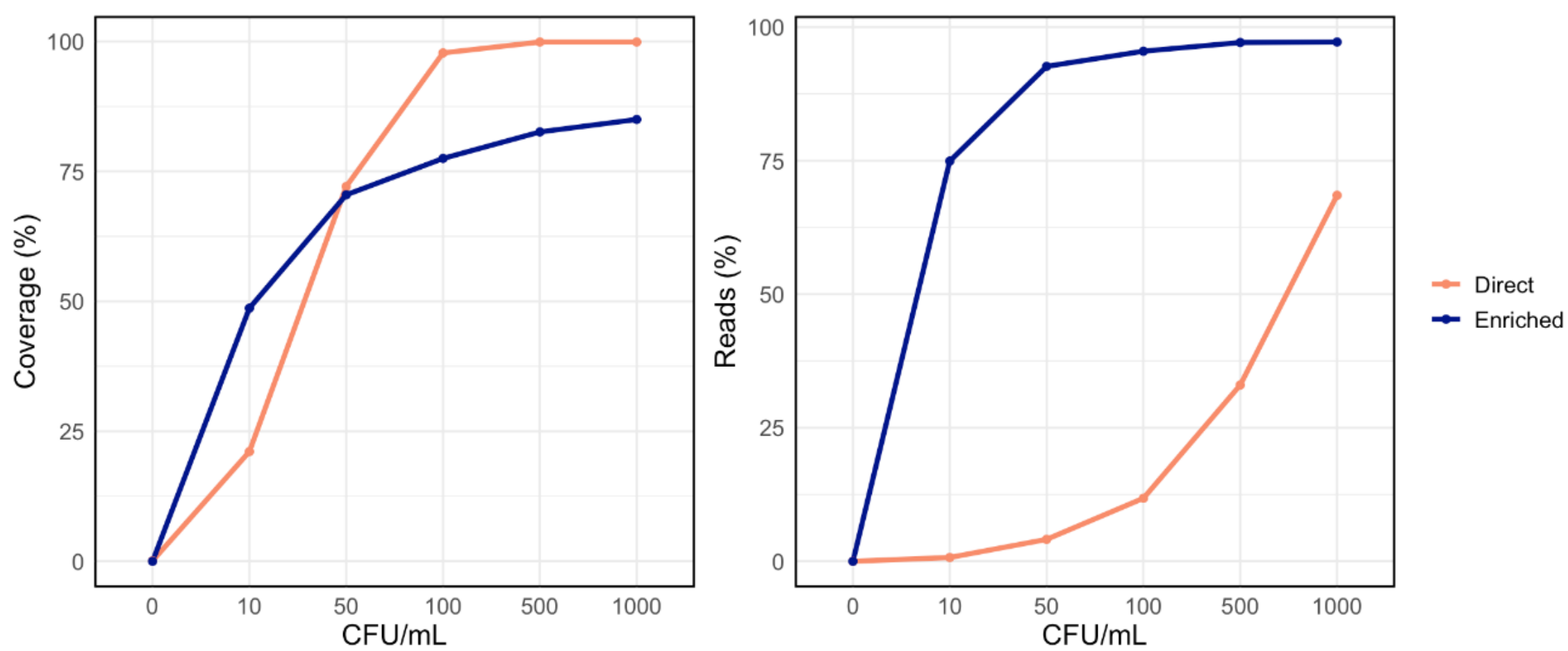

Supplementary Figure 3. Coverage of enriched and direct sequencing *Legionella* read sets across the reference genome and percentage of *Legionella* reads observed in enriched and direct sequencing read sets of validation samples.

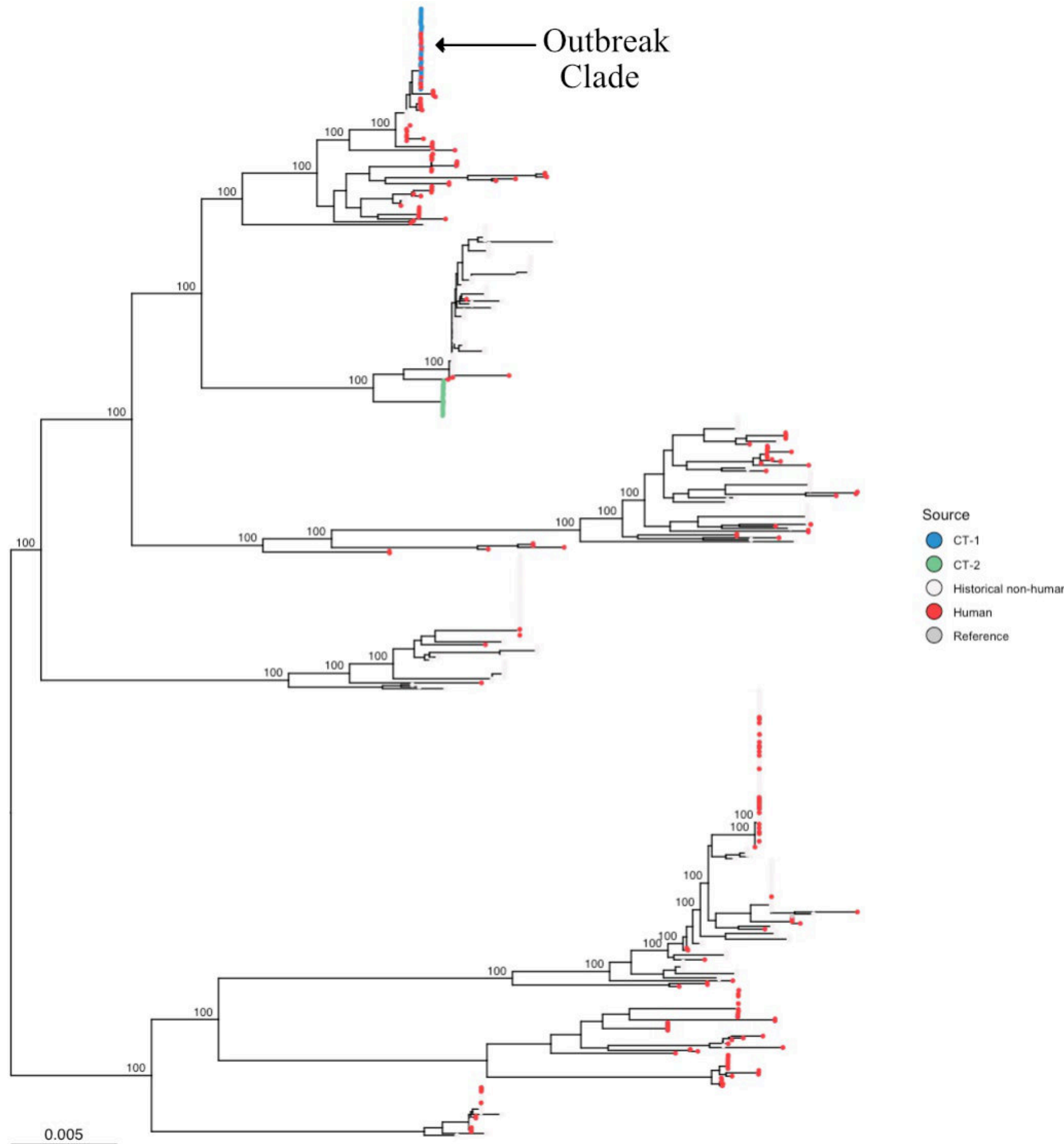

Supplementary Figure 4. Maximum-likelihood phylogeny of clinical and environmental *Legionella pneumophilla* genomes collected as part of the 2024 outbreak. The phylogeny was constructed with IQ-TREE 2 **(1)** and visualized in R v4.3.1 using the ggtree v3.12.0, treeio v1.28.0, ggplot2 v3.5.2, readr v2.1.5 and ape v5.8-1 packages. The tree shows the full analysis of 66 clinical and environmental outbreak genomes in context with 592 historical Victorian isolates received for whole genome sequencing at MDU PHL

between 2019 and 2024. Victorian reference genome AUSMDU00010536 was used as the reference genome for single nucleotide polymorphism detections utilized through snippy (2). The core genome used for the phylogeny encompasses 81% of the reference genome.

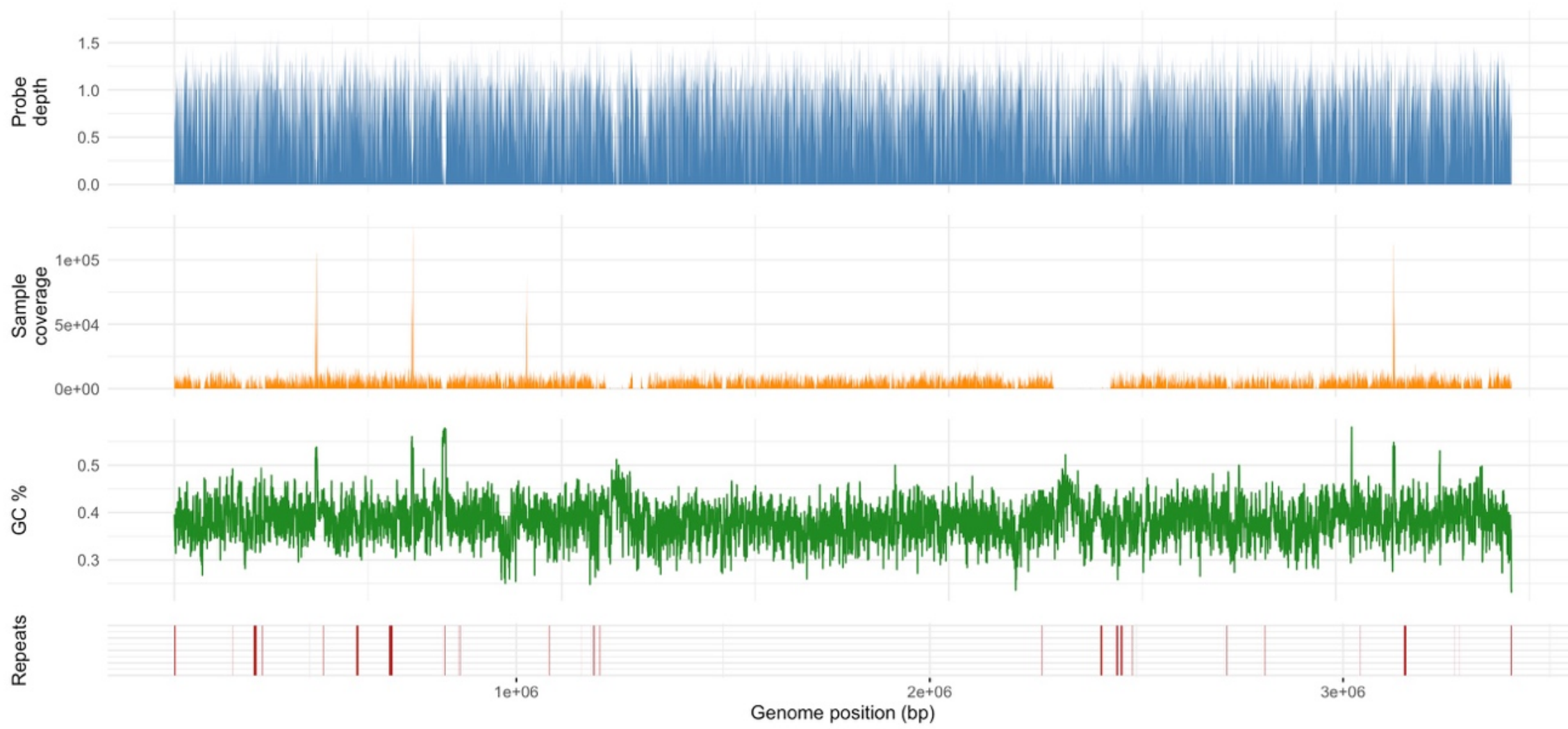

Supplementary Figure 5. Investigating regions of both increased and decreased coverage across all specimens. Tracking from top to bottom: **Probe depth** — normalized read depth of hybridization capture probes mapped across the reference genome AUSMDU000 10536 in 500 bp windows, reflecting probe density across the target region; **Sample coverage** — read depth of enrichment-sequenced outbreak isolates mapped to the reference genome, with peaks of elevated coverage (up to 1×10^5) indicative of regions with high GC content; **GC content** — percentage GC calculated in 500 bp windows across the reference genome (mean ~40%); **Repeat regions** — genomic coordinates of repetitive elements identified by self-BLAST analysis (≥90% identity, ≥200 bp). Coverage gaps observed in the sample track reflect genuine sequence divergence between outbreak isolates and the reference genome.

Supplementary Table 1. Sequence metadata and accession numbers of genomes incorporated in the data analysis. The description specifies where multiple isolates have been sequenced from a single environmental specimen or case. Data can be accessed at BioProject PRJNA941385 at the NCBI Sequence Read Archive.

Supplementary Table 2. Metadata and QC metrics of hybridization based enrichment sequencing genomes generated in this study. Read sets can be accessed can be accessed at BioProject PRJNA1426987 at the NCBI Sequence Read Archive.

Supplementary Table 3. Panel of 709 LpSG1 isolates derived from human and environmental sources across Victoria, Australia, supplemented with publicly available international genomes used for bait development.

Supplementary Table 4. Final pan-genomic bait sequences comprised of 28,061 probes.

Supplementary Table 5. Summary metrics of validation specimens. Read sets can be accessed can be accessed at BioProject PRJNA1426987 at the NCBI Sequence Read Archive.

Supplementary Table 6. List of 189 cooling towers specimens with culture and qPCR results.

Supplementary Table 7. Clusters as defined by reference free Split Kmer Analysis (SKA).

Supplementary Table 8. List of 65 environmental isolates used for model training.

Supplementary Table 9. Summary of Machine Learning model parameters.

Supplementary Table 10. List of Machine Learning probabilities table.

References

1. Minh BQ, Schmidt HA, Chernomor O, Schrempf D, Woodhams MD, von Haeseler A, Lanfear R. 2020. IQ-TREE 2: New Models and Efficient Methods for Phylogenetic Inference in the Genomic Era. Molecular Biology and Evolution 37:1530–1534.

2. Seemann T. 2020. snippy: rapid haploid variant calling and core genome alignment. https://github.com/tseemann/snippy.
